# MiR-130a regulates neurite outgrowth and dendritic spine density by targeting MeCP2

**DOI:** 10.1007/s13238-016-0272-7

**Published:** 2016-06-01

**Authors:** Yunjia Zhang, Mengmeng Chen, Zilong Qiu, Keping Hu, Warren McGee, Xiaoping Chen, Jianghong Liu, Li Zhu, Jane Y. Wu

**Affiliations:** State Key Laboratory for Brain & Cognitive Science, Institute of Biophysics, Chinese Academy of Sciences, Beijing, 100101 China; University of Chinese Academy of Sciences, Beijing, 100049 China; Laboratory of Molecular Basis of Neural Plasticity, Institute of Neuroscience, Chinese Academy of Sciences, Shanghai, 200031 China; Institute of Medicinal Plant Development (IMPLAD), Chinese Academy of Medical Sciences, Beijing, 100193 China; Department of Neurology, Center for Genetic Medicine, Lurie Cancer Center, Northwestern University Feinberg School of Medicine, Chicago, IL 60611 USA

**Keywords:** miR-130a, MECP2, neurite outgrowth, dendritic spines, dendrite morphology

## Abstract

MicroRNAs (miRNAs) are critical for both development and function of the central nervous system. Significant evidence suggests that abnormal expression of miRNAs is associated with neurodevelopmental disorders. MeCP2 protein is an epigenetic regulator repressing or activating gene transcription by binding to methylated DNA. Both loss-of-function and gain-of-function mutations in the *MECP2* gene lead to neurodevelopmental disorders such as Rett syndrome, autism and *MECP2* duplication syndrome. In this study, we demonstrate that miR-130a inhibits neurite outgrowth and reduces dendritic spine density as well as dendritic complexity. Bioinformatics analyses, cell cultures and biochemical experiments indicate that miR-130a targets *MECP2* and down-regulates MeCP2 protein expression. Furthermore, expression of the wild-type MeCP2, but not a loss-of-function mutant, rescues the miR-130a-induced phenotype. Our study uncovers the *MECP2* gene as a previous unknown target for miR-130a, supporting that miR-130a may play a role in neurodevelopment by regulating MeCP2. Together with data from other groups, our work suggests that a feedback regulatory mechanism involving both miR-130a and MeCP2 may serve to ensure their appropriate expression and function in neural development.

**Electronic supplementary material:**

The online version of this article (doi:10.1007/s13238-016-0272-7) contains supplementary material, which is available to authorized users.

## INTRODUCTION

MicroRNAs (miRNAs) are small non-coding RNAs of 16–28 nucleotides (nt) in lengths. They are transcribed into pri-miRNA, which are then cleaved by Drosha to form pre-miRNA in the nucleus. After being transported to the cytoplasm and then cleaved by Dicer, mature miRNAs incorporate with AGO proteins to form RNA-induced silencing complexes (RISCs) to silence target mRNAs (Finnegan and Pasquinelli, 2013). MicroRNAs play important roles in the formation and the function of the nervous system (McNeill and Van Vactor, 2012). Aberrant expression of miRNAs may lead to neurodevelopmental and neurodegenerative diseases (Im and Kenny, 2012). We identified microRNA-130a (miR-130a) in a search for microRNAs regulated by the Tar-DNA binding protein-43 (TDP-43) (The detail will be described in a separate paper). It has been reported that miR-130a regulates neurogenesis by inhibiting the synthesis of substance P (Greco and Rameshwar, 2007), a neuropeptide that can increase neurite outgrowth of cultured chick dorsal root ganglia (Shigehiko and Takeshi, 1978). In *Xenopus laevis*, miR-130a is expressed in anterior neural tissues, eyes and branchial arches (Walker and Harland, 2008), and down-regulation of miR-130a led to smaller eyes (Gessert et al., 2010). In mice, miR-130a is expressed in developing somites, neural tube and restricted regions of the brain after embryonic day 9.5 (Hoesel et al., 2010). Cerebral expression of miR-130a then decreases during the postnatal period throughout the rest of development and is detected only at a low level in the adult mouse cortex (Eda et al., 2011; Søe et al., 2011). Several studies suggest that dysregulation of miR-130a may be associated with neurodevelopmental disorders. For example, the expression of miR-130a is up-regulated in the serum of children with autism (Vasu et al., 2014). Analyses of the copy number variation (CNV) of miR-130a have led to the identification of 2 patients diagnosed with autism spectrum disorder carrying duplications of the locus (11q12.1) containing miR-130a (Rosenfeld et al., 2010). These reports suggest a potential role for miR-130a in neural development. However, the target genes and mechanisms by which miR-130a functions in neurons remain to be investigated.

*MECP2* (Methyl-CpG-binding Protein 2) is an X-linked gene, encoding a methylated DNA-binding protein and has been identified as a causative gene in Rett syndrome (Amir et al., 1999; Lewis et al., 1992; reviewed in Lombardi et al., 2015). The MeCP2 protein has multiple biological functions, including regulating RNA transcription, involving in RNA splicing and miRNA biogenesis (Chahrour et al., 2008; Cheng et al., 2014; Maunakea et al., 2013; Nan et al., 1998; Young et al., 2005; Lombardi et al., 2015). Loss and gain of function mutations of MeCP2 lead to Rett syndrome and *MECP2* duplication syndrome, both of which are progressive neurological disorders characterized by intellectual disability, autism and developmental regression (Lombardi et al., 2015). Postmortem brain pathology analyses, MeCP2 mouse models and *in vitro* experiments suggest that MeCP2 regulates axon outgrowth, dendritic spine formation and dendritic arbor complexity (Chao et al., 2007; Chapleau et al., 2009; Cheng et al., 2014; Jiang et al., 2013; Jugloff et al., 2005; Lombardi et al., 2015). Neuronal expression of MeCP2 at an appropriate level is crucial for neural development. However, the mechanisms by which how MeCP2 is regulated remain to be elucidated.

The mammalian *MECP2* gene has a highly conserved long 3′ untranslated region (3′UTR) which contains multiple binding sites for microRNAs (Coy et al., 1999; McGowan and Pang, 2015). It was proposed that microRNAs binding to the 3′UTR of *MECP2* and regulating its expression might contribute to neuronal maturation and neurodevelopmental diseases (Hansen et al., 2010). In the present study, we examined the interaction between miR-130a and MeCP2. Our data demonstrate that miR-130a inhibits neurite outgrowth and dendritic spine formation by regulating MeCP2.

## RESULTS

### MiR-130a is expressed in developing cerebral cortex and is predicted to regulate neuronal function

**Figure 1:**
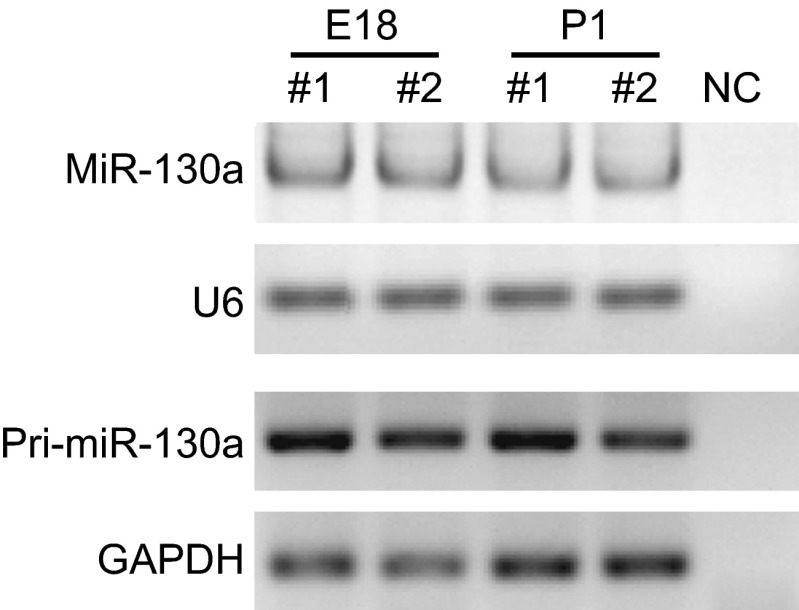
**Expression of miR-130a in embryonic and postnatal rat brains**. Rat cortical neurons were harvested from brains at the stages of E18 and P1. The levels of mature miR-130a (with U6 as an internal control) (upper panel) and primary miR-130a (with GAPDH as an internal control) (lower panel) were detected by RT-PCR. The last lane in each panel contained the negative control (NC) in which reverse transcriptase was omitted from the cDNA synthesis reactions

The mature sequence of miR-130a is conserved across various species including mammals, birds, amphibians and fish (Fig. S1). To study the function of miR-130a in neurodevelopment, we first examined the expression of miR-130a in developing rat brains. Primary and mature rno-miR-130a were detected in the rat cerebral cortices at both embryonic (E18) and postnatal (P1) stages (Fig. 1), which is consistent with previous results in mice (Hoesel et al., 2010). There were no dramatic changes of the level of miR-130a between the late embryonic and early postnatal stages.

To begin to explore biological function of miR-130a, we employed a bioinformatics approach using three miRNA target prediction tools—TargetScan, miRanda and PicTar—to predict candidate target genes for miR-130a (Agarwal et al., 2015; Krek et al., 2005; Betel et al., 2008). The pathway enrichment analysis (KOBAS 2.0; Xie et al., 2011) revealed potential target genes involved in multiple pathways important for neuronal function, such as axon guidance, synaptic vesicle formation and axonal transport. A number of the predicted miR-130a target genes are also associated with neurodevelopmental disorders, such as autism, schizophrenia and hereditary spastic paraplegia (Table 1). These results, together with its expression in brain tissues, suggest that miR-130a may play an important role in regulating expression of genes crucial for the function of the nervous system.

**Table 1:** Candidate target protein-coding genes for miR-130a

Diseases and pathways		Database	MiR-130a target genes	Gene set size	*P*-value
Neuronal function	Axon guidance	KEGG PATHWAY	9	129	0.0102891
	Synaptic vesicle formation	KEGG PATHWAY	5	63	0.0321619
	Lissencephaly gene (LIS1) in neuronal migration and development	PID	3	19	0.0403738
	SNARE interactions in vesicular transport	KEGG PATHWAY	3	36	0.0807329
Nervous system disorders	Hereditary spastic paraplegia (SPG)	KEGG DISEASE	4	21	0.0058905
	Autistic spectrum disorder	FunDO	6	68	0.013854
	Schizophrenia	FunDO	10	172	0.0239614
	Lissencephaly	KEGG DISEASE	2	6	0.0218963
	Non-syndromic X-linked mental retardation	KEGG DISEASE	3	22	0.0368521
	Congenital disorders of development	KEGG DISEASE	13	244	0.0426269
	Isolated orofacial clefts	KEGG DISEASE	2	14	0.0803631
	Spinocerebellar ataxias	FunDO	2	12	0.0520478

Target genes for miR-130a were predicted using TargetScan, miRanda and PicTar. The intersection of the results from all three tools was accepted as the potential targets. A total of 475 potential target genes were identified by the bioinformatics analyses (see MATERIALS AND METHODS). Shown in the table are the pathways most enriched with protein-coding genes predicted to be targets of miR-130a (“MiR-130a target genes” column) and corresponding raw *P*-values. The “Gene set size” is determined based on the protein coding genes in the genome that have been annotated with the specific pathways by the respective database.

### MiR-130a inhibits neurite outgrowth

**Figure 2:**
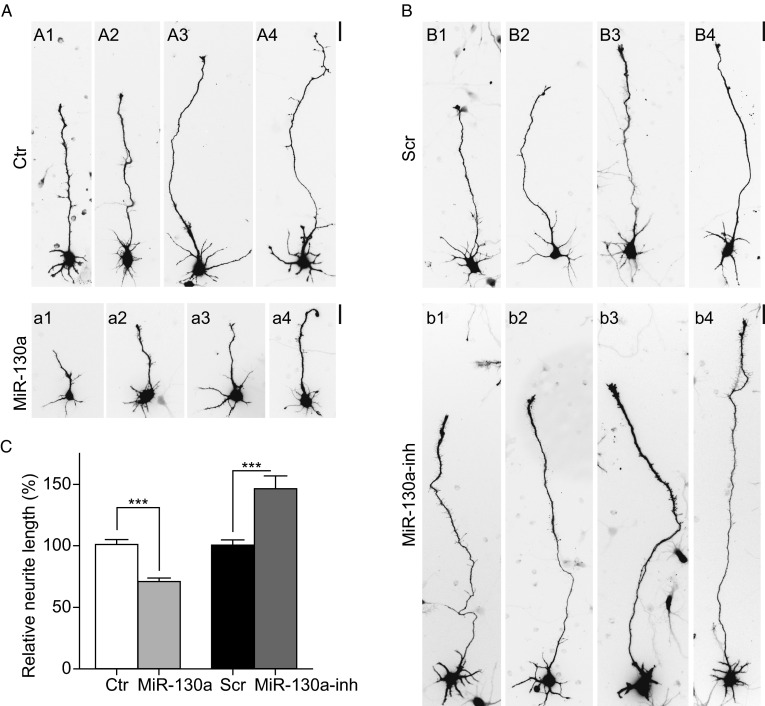
**MiR-130a decreases the length of neurites in cultured primary cortical neurons**. (A) Cortical neurons isolated from E18 rat embryos were electroporated with the vector control (Ctr; upper panels A1–A4) or the miR-130a expression plasmid (lower panels a1–a4) and cultured for 3 days. Representative images of neurons are shown from either control or miR-130a groups to illustrate the range of neurite outgrowth. Scale bar: 25 μm. (B) Cortical neurons from E18 embryos were electroporated with the scrambled RNA control (Scr; upper panels B1–B4) or the miR-130a inhibitor (MiR-130a-inh; lower panels b1–b4) and cultured for 3 days. Representative images of neurons are shown for the corresponding groups to illustrate the range of neurite outgrowth. Scale bar: 25 μm. (C) Quantification of the average lengths of the longest neurites (mean values ± SEM) in experiments shown in panels (A) and (B). At least 100 neurons in each group were randomly selected and scored in each experiment. ***, *P* < 0.001, Mann-Whitney test. Data represent three independent experiments

Neuronal differentiation is an early and fundamental event in neurodevelopment. The sprouting and outgrowth of neurites followed by the formation of axons and dendrites is an initial critical process in the early stage of development of the nervous system (Lefebvre, Sanes and Kay, 2015; Takano et al., 2015 and references within). To investigate the role of miR-130a in neuronal development, we examined the effect of miR-130a on neurite outgrowth using primary rat neuronal cultures. Cortical neurons were transfected with a control vector or a plasmid expressing pri-miR-130a that also expressed GFP to mark transfected neurons. Seventy-two hours following transfection, the neurite length was measured by tracing the longest neurite of randomly-selected transfected neurons and quantified using Image J, as published previously (Gao et al., 2010 and reference within). Neurons transfected with miR-130a showed shorter neurite length, approximately 65% of that in the control neurons (Fig. 2A and 2C). To confirm the effect of miR-130a on neurite outgrowth, a specific RNA inhibitor for miR-130a was used. Rat primary cortical neurons were transfected with a scrambled RNA control or the miR-130a inhibitor together with a plasmid expressing GFP. Neurons transfected with the miR-130a inhibitor showed greater neurite length, approximately 150% of that in neurons transfected with the control scrambled RNA (Fig. 2B and 2C). This effect of miR-130a on the neurite length was not due to its effect on neuronal survival, because miR-130a did not affect neuronal death as shown by a Terminal deoxyribonucleotidyl transferase-mediated dUTP-digoxigenin nick end labeling (TUNEL) assay and nuclear staining (Fig. S2). We also examined whether miR-130 affected the number of neurite branches per neuron (Fig. S3A). Quantitative analyses of distribution of neurite branch numbers did not show any significant difference between the control and miR-130a groups, indicating that neurite numbers were not affected by miR-130a (Fig. S3B).

### MiR-130a expression decreases dendritic complexity

**Figure 3:**
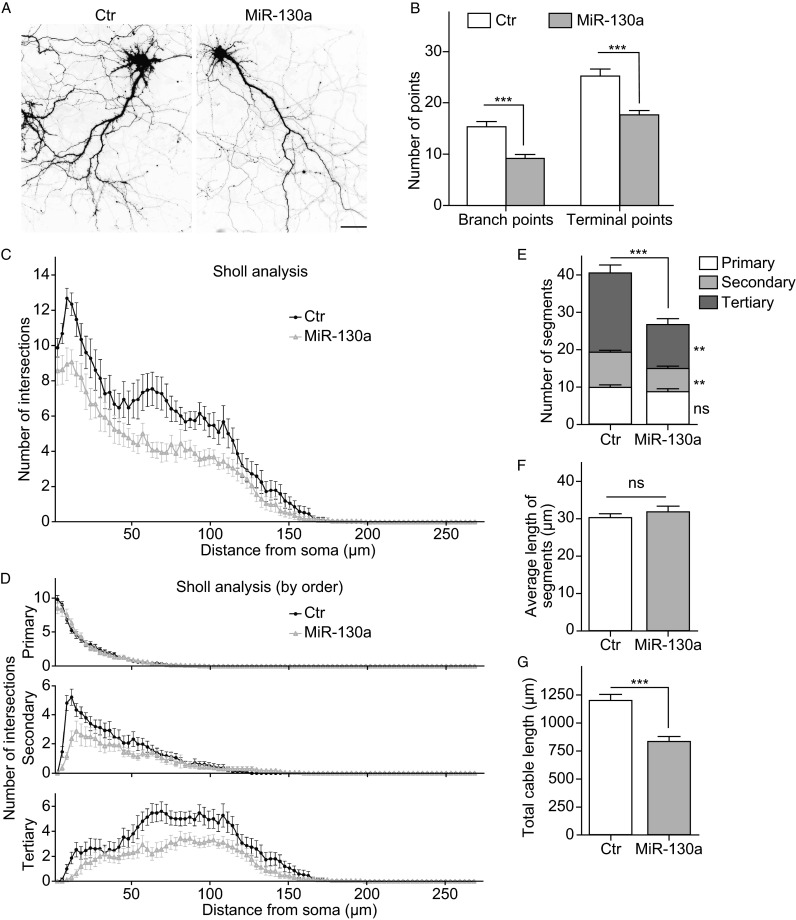
**MiR-130a decreases the complexity of dendrites**. (A) Cortical neurons were cultured for 7 days and transfected with control or miR-130a. Cells were fixed at 18 DIV. Images were taken using Leica confocal SP8. Image of 40× microscopy were demonstrated. Scale bar: 25 μm. (B) The number of branch points and terminal points per cell. (C) The Sholl analysis of dendritic complexity as measured by number of intersections. (D) The Sholl analysis of dendritic complexity by branch order-primary, secondary and tertiary branches. (E) The number of segments in total and by branch order. (F) Average length of segments per cell. (G) The total length of branches per cell. **, *P* < 0.01; ***, *P* < 0.001, Mann-Whitney test. Fifteen neurons were scored in each group in each experiment. The bar diagram represents mean values ± SEM. Data represent 3 independent experiments

Dendritic formation and maturation is critical for the formation and maintenance of the nervous system. To study the effect of miR-130a on dendrite formation, rat E18 primary cortical neurons were cultured for 7 days and then transfected with the control or miR-130a, and then fixed at DIV18 for examining dendritic morphology. The dendrites were traced and analyzed using NeuronJ (ImageJ) and NeuronStudio with neuronal morphology quantified using Sholl analysis as described previously (Kutzing et al., 2010; Langhammer et al., 2010). Dendritic complexity was decreased in miR-130a transfected neurons as compared with the control neurons, including the number of branch points and terminal points per neuron, as well as the number of intersections (Fig. 3A–C). Further analyses revealed that miR-130a decreased the number of secondary and tertiary branches, but did not affect primary branches (Fig. 3D and 3E). The lack of effect on primary branches by miR-130a is consistent with what was observed with the number of neurites (Fig. S3). In addition, the total length of dendrites was decreased in miR-130a expressing neurons as compared with the control group, although the average length of segments did not change (Fig. 3F and 3G). These data together show that miR-130a expression reduces dendritic complexity, in particular, inhibits the formation of the secondary and tertiary branches.

### *MECP2* is a target of miR-130a

**Figure 4:**
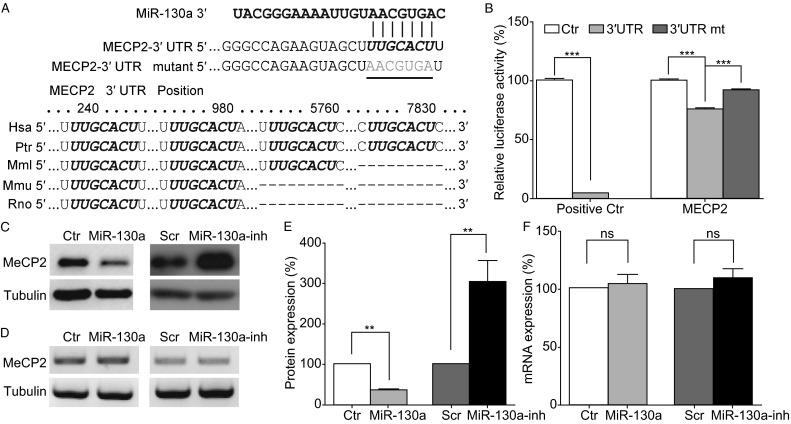
**The** ***MECP2*** **gene is a target of miR-130a**. (A) Alignment of the binding sites of miR-130a on MECP2 mRNA 3′UTR. The seed regions of miR-130a binding sites are in italics. Luciferase reporter plasmids containing the human MECP2-3′UTR regions, either the wild type UTR (MECP2-3′UTR) or the mutant UTR with the most highly conserved binding site for miR-130a mutated as shown (MECP2-3′UTR mutant). (B) A dual luciferase reporter assay. Relative luciferase activity was measured in HEK293 cells at 48 h post-transfection. The wild-type MECP2-3′UTR is referred to as “3′UTR”, whereas the mutant described in panel (A) is indicated as “3′UTRmt”. (C) The MeCP2 protein levels as detected by Western blotting. Rat E18 cortical neurons were electroporated with the control vector, miR-130a, scramble RNA (Scr) or miR-130a inhibitor (miR-130a-inh). β-Tubulin was used as an internal control. (D) The MeCP2 mRNA levels as assayed by RT-PCR with β-tubulin as an internal control. (E and F) Quantification and statistical analysis of data in (C) and (D). The bar diagram represents mean values ± SEM. Data represent three independent experiments. *, *P* < 0.05; **, *P* < 0.01; ***, *P* < 0.001. Mann-Whitney test

To investigate mechanism of miR-130a function, we sought to identify candidate target genes for miR-130a. Among the potential target gene pathways predicted by our bioinformatics analyses (Table 1), we decided to first examine candidate genes involved in neurodevelopment and associated with neurodevelopmental diseases. *MECP2* is one of these predicted target genes for miR-130a. The human *MECP2* gene has a long conserved 3′UTR (~8 kb) with containing 4 predicted miR-130a binding sites. The most upstream miR-130a binding site located ~240 nucleotide downstream of the stop codon is highly conserved, from human to rodents (Fig. 4A). To validate that *MECP2* is a target gene of miR-130a, a dual luciferase reporter assay in HEK293 cells was performed. A luciferase reporter plasmid was constructed containing a 415-bp fragment of the 3′UTR of the human *MECP2* gene including the most highly conserved miR-130a binding site (the most upstream binding site in the 3′UTR; Fig. 4A). When co-transfected with miR-130a, luciferase activity of the *MECP2* 3′UTR reporter was significantly reduced, whereas mutating the predicted miR-130a binding site in the *MECP2* reporter (3′UTRmt) reversed the miR-130a inhibition, indicating that the 3′UTR of the human *MECP2* gene is responsive to miR-130a expression (Fig. 4B). The mRNA and protein levels of MeCP2 were examined following transfection of pri-miR-130a or miR-130a inhibitor. Western blotting of transfected rat cortical neurons showed expression of miR-130a decreased MeCP2 protein expression to ~40% of that in the control group, whereas the expression of the miR-130a inhibitor increased the MeCP2 protein level to ~3 fold of that in the scramble control group (Fig. 4C and 4E). RT-PCR assays showed that expression of neither miR-130a nor miR-130a inhibitor altered the mRNA levels of MeCP2 (Fig. 4D and 4F). Because miRNAs may regulate protein expression by translational inhibition or mRNA degradation or both mechanisms (Hausser and Zavolan, 2014; Vidigal and Ventura, 2015; Wilczynska and Bushell, 2015), these results suggest that miR-130a may decrease the MeCP2 protein level by affecting translation efficiency rather than degrading MeCP2 mRNA. Future studies are necessary to elucidate the precise mechanisms by which miR-130a regulates MeCP2 protein production.

### Restoring MeCP2 expression rescues miR-130a-mediated inhibition of neurite outgrowth

**Figure 5:**
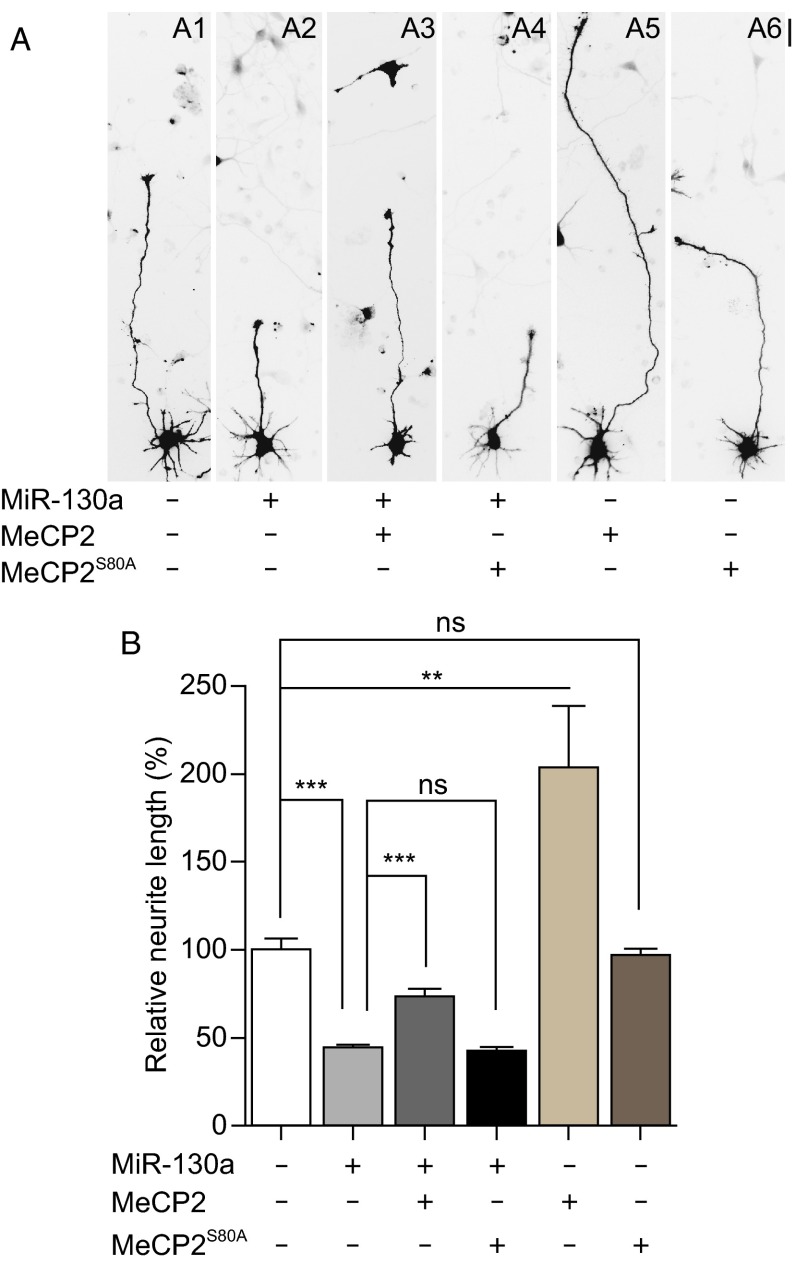
**Expression of miR-130a decreases the length of neurites, a phenotype partially rescued by wild type MeCP2 but not by a loss-of-function MeCP2 mutant**. (A) Rat E18 cortical neurons were electroporated with the vector control, miR-130a, MeCP2, MeCP2^S80A^ or miR-130a together with MeCP2 or MeCP2^S80A^ and cultured for 3 days before imaging. Scale bar: 25 μm. (B) The average length of the longest neurites in (A) were measured and statistically analyzed from three independent experiments (mean values ± SEM; at least 100 neurons in each group were scored in each experiment). **, *P* < 0.01; ***, *P* < 0.001. Mann-Whitney test

It was reported that MeCP2 promotes axon outgrowth in mouse primary cortical neurons (Jugloff et al., 2005). The phosphorylation of MeCP2 at Ser80 is necessary for MeCP2 chromatin binding ability and neurological function (Tao et al., 2009). When the Ser80 was mutated to alanine (MeCP2^S80A^), the S80A-mutant MeCP2 protein cannot be phosphorylated, acting as a loss-of function mutant. We co-transfected the wild-type MeCP2 or MeCP2^S80A^ together with miR-130a into cortical neurons and examined if MeCP2 could rescue the effect of miR-130a on the neurite outgrowth. The average neurite length was measured as described above. Neurons transfected with MeCP2 alone showed longer neurite length, approximately 2 fold of that of control neurons, whereas neurons transfected with MeCP2^S80A^ mutant did not show any changes in the average neurite length (Fig. 5A and 5B). Expression of MeCP2 in miR-130a transfected neurons significantly increased neurite length, ~30% longer than that of neurons transfected with miR-130a alone. However, expression of the mutant form of MeCP2, MeCP2^S80A^, ablated this rescue effect. Our data show that miR-130a negatively regulates neurite length in part by down-regulating MeCP2.

### MiR-130a decreases dendritic spine density and MeCP2 rescues the miR-130a-induced phenotype

**Figure 6:**
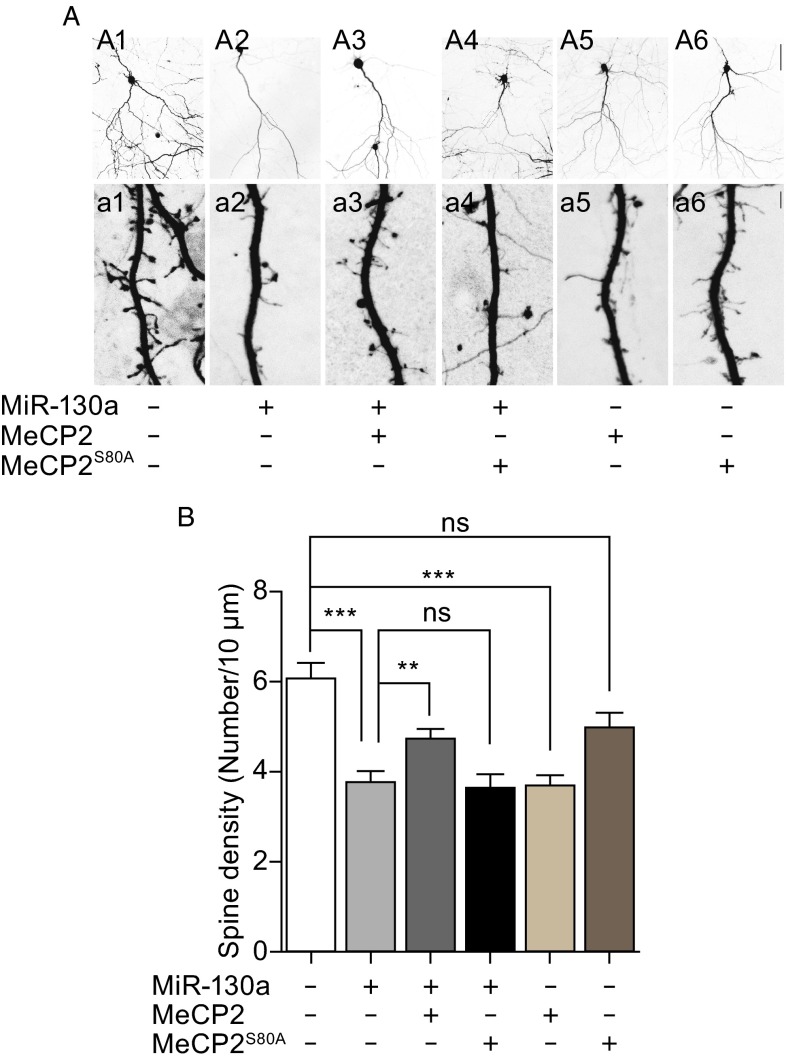
**Expression of miR-130a decreased the density of dendritic spines, a phenotype that can be rescued by functional MeCP2 but not by MeCP2 mutant**. (A) Rat E18 cortical neurons were cultured for 21 days and then transfected with vector control, miR-130a, MeCP2, MeCP2^S80A^ or miR-130a mixed with MeCP2 or MeCP2^S80A^. Upper panels (A1–A6): image of 20× microscopy demonstrated the healthy morphology of neurons. The boxed areas by dotted lines in the top panels are enlarged and shown in lower panels and used for quantification of the secondary and tertiary dendrites. Scale bar: 75 μm. Lower panels (a1–a6): high magnifications of image (100× lens; zoom 4.0×) demonstrated the morphology of dendritic spines. Scale bar: 2.5 μm. (B) The average density of dendritic spines. Spines on the secondary and tertiary dendrites were measured. The bar diagram represents mean values ± SEM. Data represent three independent experiments (Ten neurons were scored in each experiment). **, *P* < 0.01; ***, *P* < 0.001, Mann-Whitney test

Dendritic spines are membrane protrusions on dendrites and play critical roles in the formation of synapses and neural circuits (Ethell and Pasquale, 2005). We examined the effect of miR-130a on dendritic spine formation. In this set of experiments, rat E18 primary cortical neurons were cultured for 21 days *in vitro* and then transfected using Lipofectamine with the control or with miR-130a. Cells were then fixed 3 days post-transfection. The density of dendritic spines on the secondary and tertiary dendritic branches was determined. Neurons transfected with miR-130a showed fewer dendritic spines as compared with the control neurons (Fig. 6A, panel a1 and a2). Both gain-of- and loss-of-function mutations of the *MECP2* gene lead to neurodevelopment disorders with dendritic spine morphological abnormalities, suggesting the proper level of functional MeCP2 is critical for neural development (Jiang et al., 2013; Xu et al., 2014). Indeed, overexpression of MeCP2 alone reduced dendritic spine density (Fig. 6A, panel a5), consistent with previous data from hippocampal slice cultures (Xu et al., 2014).

We then asked whether the phenotype of decreased spine density by miR-130a was mediated by regulating MeCP2. Neurons co-expressing miR-130a and MeCP2 showed a partial rescue effect, with a higher density than neurons transfected with either miR-130a or MeCP2 alone (Fig. 6A, panel a3). This rescue effect was not observed when S80A mutant MeCP2 was co-transfected with miR-130a (Fig. 6A, panel a4). Taken together, our data suggest that miR-130a decreases dendritic spine density in part by regulating MeCP2.

## DISCUSSION

In this study we sought to determine the role of miR-130a in neuronal development, and examined whether effects of miR-130a were mediated by regulating *MECP2* gene expression. Consistent with previous studies carried out in mice (Hoesel et al., 2010; Eda et al., 2011; Søe et al., 2011), miR-130a is expressed in rat cortical neurons at the late embryonic and early postnatal stages (Fig. 1). Bioinformatics analyses suggest that miR-130a may be involved in neurogenesis and associated with neurodevelopmental disorders such as schizophrenia, lissencephaly and autism (Table 1) (Poluch and Juliano, 2015; Selemon and Zecevic, 2015; Wang and Baraban, 2007; Wegiel et al., 2010). Neurogenesis is not only essential for the formation of the nervous system during development, but also critical for the maintenance and neural repair in the adult brain (Feliciano et al., 2015; Zhao et al., 2008). Defects in neurogenesis are associated with a large number of neurological disorders, including neurodevelopmental diseases and neurodegenerative disorders such as Alzheimer’s Disease and Parkinson’s Disease (Merson and Bourne, 2014; Schoenfeld and Cameron, 2015; Winner and Winkler, 2015).

Our bioinformatics analyses suggest that miR-130a may be involved in many pathways critical for neurodevelopment and associated with pathogenesis of neurodevelopmental diseases (Table 1). Using primary neuronal cultures, we provided experimental evidence that miR-130a plays a role in regulating both neurite outgrowth and dendritic formation. The outgrowth of neurites, which will differentiate into axons and dendrites, is an important event in neural development (Takano et al., 2015; Lefebvre, Sanes, Kay 2015). MiR-130a expression reduces neurite outgrowth, whereas miR-130a inhibitor increases neurite outgrowth (Fig. 2). Expression of miR-130a also reduces dendritic complexity without affecting the number of primary dendritic branches (Fig. 3). Finally, miR-130a reduces the density of dendritic spines (Fig. 6). Overall, these results suggest that miR-130a may play a complex role in regulating neurogenesis and neuronal differentiation. The expression of miR-130a was reported to be decreased in the cerebral cortex of mouse brain but increased in hippocampus from birth to adulthood (Eda et al., 2011). This dynamic expression pattern of miR-130a suggests that the function of miR-130a in the nervous system may be different depending on the specific regions and developmental stages. It should be noted that it has been observed that the expression of a number of miRNAs was affected in MeCP2-deficient mice, including the down-regulation of miR-130a (Urdinguio et al., 2010). Together with our data, these results suggest that miR-130a together with MeCP2 may participate in a feedback regulatory loop that maintain the appropriate levels of both miR-130a and MeCP2 to ensure their proper function of neural development in a spatially and temporally regulated manner. It is obvious that further studies are necessary for elucidating the role of miR-130a in neurodevelopment.

Our data support that miR-130a inhibits neurite outgrowth, at least in part, by regulating *MECP2*, a predicted target gene by three bioinformatics tools. MiR-130a expression decreased the protein level of MeCP2 but did not affect its mRNA level (Fig. 4). This can be explained by the possible activity of miR-130a in regulating translation efficiency rather than mRNA stability, a mechanism that has been described for a subset of miRNAs (Baek et al., 2008). The human *MECP2* gene has at least 4 transcripts of different lengths, among which the 10 kb mRNA contains the longest 3′UTR (~8 kb). This transcript is predominantly expressed in the brain, whereas the short transcripts are mainly expressed in the lung and liver (Pelka et al., 2005). Only the longest 3′UTR contains multiple miR-130a binding sites in different species. In addition, the 3′UTR of *MECP2* is highly conserved in both sequence and RNA secondary structure (Coy et al., 1999). These all suggest that miR-130a may regulate MeCP2 in a conserved manner and the regulation may be relatively specific to the nervous system. Variants in the *MECP2 * 3′UTR were found among patients affected by autism and Rett syndrome, although none of the variants is predicted to be pathogenic to Rett syndrome (Santos et al., 2008; Shibayama et al., 2004). However, MeCP2 mRNA levels in four autism patients carrying conserved 3′UTR alterations were lower (Coutinho et al., 2007). These findings suggest that mutations in the 3′UTR might impact the expression of MeCP2 (McGowan and Pang, 2015).

It has been reported previously that DNA methylation and histone modification are important to neurite outgrowth in PC12 cells (Futamura et al., 1995; Persengiev and Kilpatrick, 1996). MeCP2 is a methylated DNA binding protein regulating gene transcription, playing important roles in histone deacetylation and chromatin remodeling by recruiting histone deacetylase (Chahrour et al., 2008; Jones et al., 1998; Nan et al., 1998). MeCP2 was reported to promote axon outgrowth in mouse cortical neurons, and the suppression of MeCP2 in PC12 cells inhibited the neurite extension (Cusack et al., 2004; Jugloff et al., 2005). MeCP2^S80A^ could not be phosphorylated and lacked the chromatin binding ability (Tao et al., 2009). In addition, it was found that S80-phosphorylated MeCP2 could suppress nuclear miRNA processing by binding Drosha, the initial processing complex for generating pre-miRNAs, and regulate Drosha function in an activity-dependent manner (Cheng et al., 2014). In our study, the wild type MeCP2 promoted neurite outgrowth and effectively rescued the phenotype induced by miR-130a, whereas MeCP2^S80A^ lost this activity (Fig. 5). Because MeCP2 also regulates microRNA biogenesis, including that of miR-130a (Urdinguio et al., 2010), it might be difficult to interpret complex results if the rescue experiments were performed in MeCP2-null background. These results suggest that miR-130a inhibits neurite outgrowth by targeting MeCP2, and that MeCP2 may affect neurite outgrowth via its activity in regulating chromatin structure and/or modulating miRNA processing. Much more work is necessary to further understand the underlying mechanisms, especially the reciprocal relationship between MeCP2 and miR-130a.

Reduced dendritic spine density is a common feature in individuals with Rett syndrome (Phillips and Pozzo-Miller, 2015; Xu et al., 2014), knockdown of the endogenous MeCP2 also reduced dendritic spine density (Chapleau et al., 2009). Consistent with this, expression of MeCP2 rescued the decreased spine density caused by miR-130a. It has been reported that dendritic spine density in terminal dendritic branches of young transgenic mice expressing MeCP2 (a *MECP2* duplication syndrome mouse model) was initially higher than the control group and then decreased after postnatal week 12 (Jiang et al., 2013). The dendrite outgrowth phenotype in the MeCP2 overexpressing mice during the early stage (See Fig. 1 of Jiang et al., 2013) is consistent with our observation. On the other hand, it has also been reported that expression of MeCP2 inhibited dendritic spine development in rat hippocampal slice cultures (Cheng et al., 2014). Data from different studies suggest that both MeCP2 overexpression and MeCP2 loss of function result in complex phenotypes in dendritic development (Na et al., 2013; Zhou et al., 2006; Jiang et al., 2013; Cheng et al., 2014). Therefore, the observation in our neuronal culture system that MeCP2 increased dendritic spine density in miR-130a expressing neurons may reflect a delicate balance and dynamic changes in the combinatorial effects of MeCP2 and microRNAs during dendritic development.

Neurons require proper homeostasis of MeCP2 to function normally (Lombardi et al., 2015). Loss of function of MeCP2 leads to severe neurodevelopmental diseases such as Rett syndrome, autism and schizophrenia; whereas the gain of function of MeCP2 may cause *MECP2* duplication syndrome (Lombardi et al., 2015; Van Esch, 2011). Phenotypes of Rett syndrome and *MECP2* duplication syndrome in mouse models can be reversed if MeCP2 level is properly restored (Robinson et al., 2012; Sztainberg et al., 2015). Taken together, our data support that miR-130a exhibits an inhibitory role on neurite outgrowth, dendritic branching and dendritic spine maturation, partially by targeting MeCP2. Seminal reviews on miRNAs have discussed about miRNAs as multi-facet regulators acting beyond simple repression of gene expression: buffering noise in the expression of their targets or setting a threshold-linear response (Bartel, 2009; Hausser and Zavolan, 2014). In this way, miR-130a could serve to buffer changes in MeCP2 protein expression and may help maintain MeCP2 at an appropriate level, which is essential for its function. Future studies are necessary to elucidate the complex mechanisms controlling all these events.

## MATERIALS AND METHODS

### Prediction of target genes for miR-130a by bioinformatics analyses

Target genes for miR-130a were predicted using TargetScan, miRanda and PicTar. The intersection of the results from all three tools was accepted as the potential targets. The Entrez Gene IDs of potential targets were downloaded from Ensembl and entered into KOBAS2.0 (with default setting) for annotation of gene pathways, disease associated genes and GeneOntology (GO) terms. The result of annotation was then used to identify statistically significantly enriched pathways, diseases and GO terms using protein-coding genes from the whole human genome set (20,192 genes) as background.

### Cell culture and transfection

Embryonic day 18 (E18) rat cortical neurons were cultured and transfected following published protocols (Gao et al., 2010; Guo et al., 2011). Briefly, following electroporation (Amaxa Nucleofector II), neurons were seeded onto poly-D-lysine coated coverslips in 12-well plates. In the experiments for analyses of dendrite morphology and spine density, Lipofectamine2000 was used for transfecting neurons at 7 days and 21 days *in vitro* (DIV).

### Plasmids and RNA inhibitors

MeCP2 and MeCP2^S80A^ plasmids were as described previously (Cheng et al., 2014). MiR-130a plasmid was generated using pCDH-CMV-MCS-EF1-copGFP (CD511B-1, System Biosciences) according to the manufacturer’s instructions. MiR-130a genomic sequence was amplified with the following primers: forward, 5′-TGCTCTAGAGGTCATCTGAGAGTGTTGCCT-3′; reverse, 5′-CCGGGATCCTGACCCTCAGTTTTTCATCCA-3′. The miR-130a inhibitor was a methylation-modified RNA oligonucleotide complementary to miR-130a (GenePharma).

### Luciferase reporter assay

Plasmids expressing wild-type or mutant human *MECP2* 3′UTR reporter genes containing a 415-bp fragment including the most conserved miR-130a binding site (shown as the “240 site” in Fig. 4A) were constructed using psiCHECK2 vector (Promega). The PCR amplification primers were as follows, wild-type MECP2-3′UTR primers: forward primer, 5′-CCGCTCGAGCGGAGCGGATTGCAAAGC-3′; and reverse primer, 5′-ATTTGCGGCCGCTGTAGACGGGGCACTGATGG-3′. The mutant forward primer is designed across the first miR-130a binding site, introducing a mutation that scrambles the entire seed region (underlined portion, see Fig. 4A): MECP2-3′UTR mutant primers: forward, 5′-GGCCAGAAGTAGCTAACGTGATTTCTA AACTAGGCTC-3′; and reverse, 5′-GCCTAGTTTAGAAATCACGTTAGCTACTTCTGGCCC-3′. The positive control was the same vector inserted with the complementary sequence of miR-130a. Luciferase assay was performed using Dual-Luciferase® Reporter Assay System (Promega). MiR-130a and MECP2 3′UTR were co-transfected to HEK293 cells at the ratio of 36.5:1. 48 h following transfection, cells were lysed with the activity of Renilla luciferase measured according to the manufacturer’s instructions.

### RNA extraction and RT-PCR

Total RNA was extracted using TriZol reagent (Invitrogen). Genomic DNA was removed with RNase-free DNase (NEB). Total cDNA was reverse-transcribed using SuperScript™ III Reverse Transcriptase (Invitrogen). PCR primers of MeCP2 are: forward, 5′-AGAGGAAGTCTGGTCGCTCT-3′, reverse, 5′-CCAGGCTTTCTACCCCGTTT-3′. Primary miR-130a: forward, 5′-AGGATGAGAGGAAGGCTGTG-3′, reverse, 5′-AGAAAACAGTGACGCTGAGG-3′; GAPDH: forward, 5′-CCCCCAATGTATCCGTTGTG-3′, reverse, 5′-TAGCCCAGGATGCCCTTTAGT-3′; U6: forward, 5′-CTCGCTTCGGCAGCACA-3′, reverse, 5′-AACGCTTCACGAATTTGCGT-3′.

RT-PCR detection of mature miR-130a was performed using poly(T) adaptor-PCR as described previously (Shi et al., 2012). Poly (T) adaptor: 5′-AAGCAGTGGTATCAACGCAGAGTGC(T)_30_VN-3′; poly(T) adaptor reverse primer: 5′-CTCACACGACTCACGACAGGGCAAGCAGTGGTATCAACGCAGAGTG-3′; miR-130a specific forward primer: 5′-TGCGGCAGTGCAATGTTAAAAGGGCAT-3′.

### TUNEL assay

E18 rat cortical neurons were dissociated and electroporated with control vector or miR-130a plasmid. Cells were cultured for 3 days. Terminal deoxyribonucleotidyl transferase (TDT)-mediated dUTP-digoxigenin nick end labeling (TUNEL) was performed using In Situ Cell Death Detection Kit (Roche) as described in (Zhu et al., 2014).

### Western blotting analysis and Immunostaining

MeCP2 antibody was prepared as previously described (Hu et al., 2006). Tubulin antibody and secondary antibodies were from ProteinTech Group. Protein lysates were prepared from rat brain tissues or cultured cells for Western blotting analyses using corresponding antibodies following our previously published protocols with a chemiluminescence HRP detection kit (Millipore) (Guo et al., 2011; Deng et al., 2015).

For immunostaining, cells were washed in pre-warmed phosphate-buffered saline (PBS) and fixed in 4% paraformaldehyde (PFA). The fixed cells were permeabilized and blocked with 10% normal goat serum (NGS) plus 0.3% Triton X-100 in PBS, incubated with primary antibody: rabbit anti-beta tubulin antibody (1:70) (PTG). After washing 5 times, the cells were incubated with secondary antibody conjugated to Alexa Fluor® 594 (1:500) (Invitrogen) and stained with 4’,6’-diamidino-2-phenylindole (DAPI; 1 μg/mL). The coverslips were mounted with PermaFluor aqueous mounting medium (Thermo).

### Morphological analyses of axons and dendrites

The images for neurite outgrowth were taken using an inverted fluorescence microscope (Nikon) following a previously published protocols (Gao et al., 2010). The images for dendrite morphology and dendritic spines were taken using a confocal microscope (Leica SP8); image analyses were carried out following the published protocols (Langhammer et al., 2010; Srivastava et al., 2011).

### Statistical analyses

The data was normalized to the control group. Statistical analyses were performed using Mann-Whitney test, between individual groups and two-way ANOVA for comparing distributions. *, *P* < 0.05; **, *P* < 0.01; ***, *P* < 0.001. The bar diagram represents mean values ± SEM.

## Electronic supplementary material

Below is the link to the electronic supplementary material.

Supplementary material 1 (TIFF 179 kb)

Supplementary material 2 (TIFF 2321 kb)

Supplementary material 3 (TIFF 986 kb)
