# Supplementary figures and images for: MiR-130a regulates neurite outgrowth and dendritic spine density by targeting MeCP2
Source: Protein Cell. 2016 Jun 1;7(7):489–500. doi: 10.1007/s13238-016-0272-7 (PMC4930766; doi:10.1007/s13238-016-0272-7)

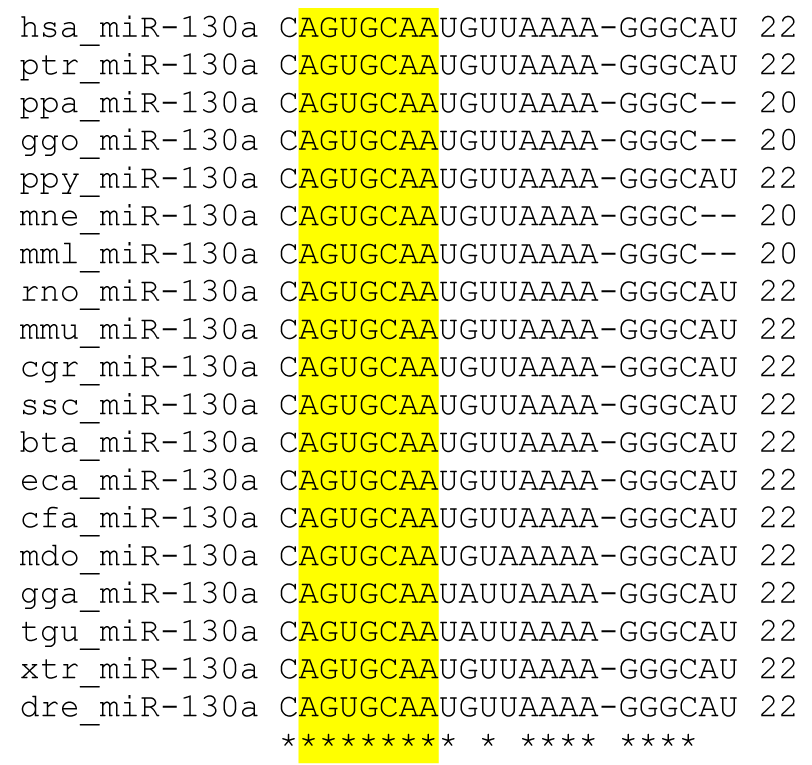

Supplement: Supplementary file 1 — Supplementary material 1 (TIFF 179 kb) [file 13238_2016_272_MOESM1_ESM.tif]

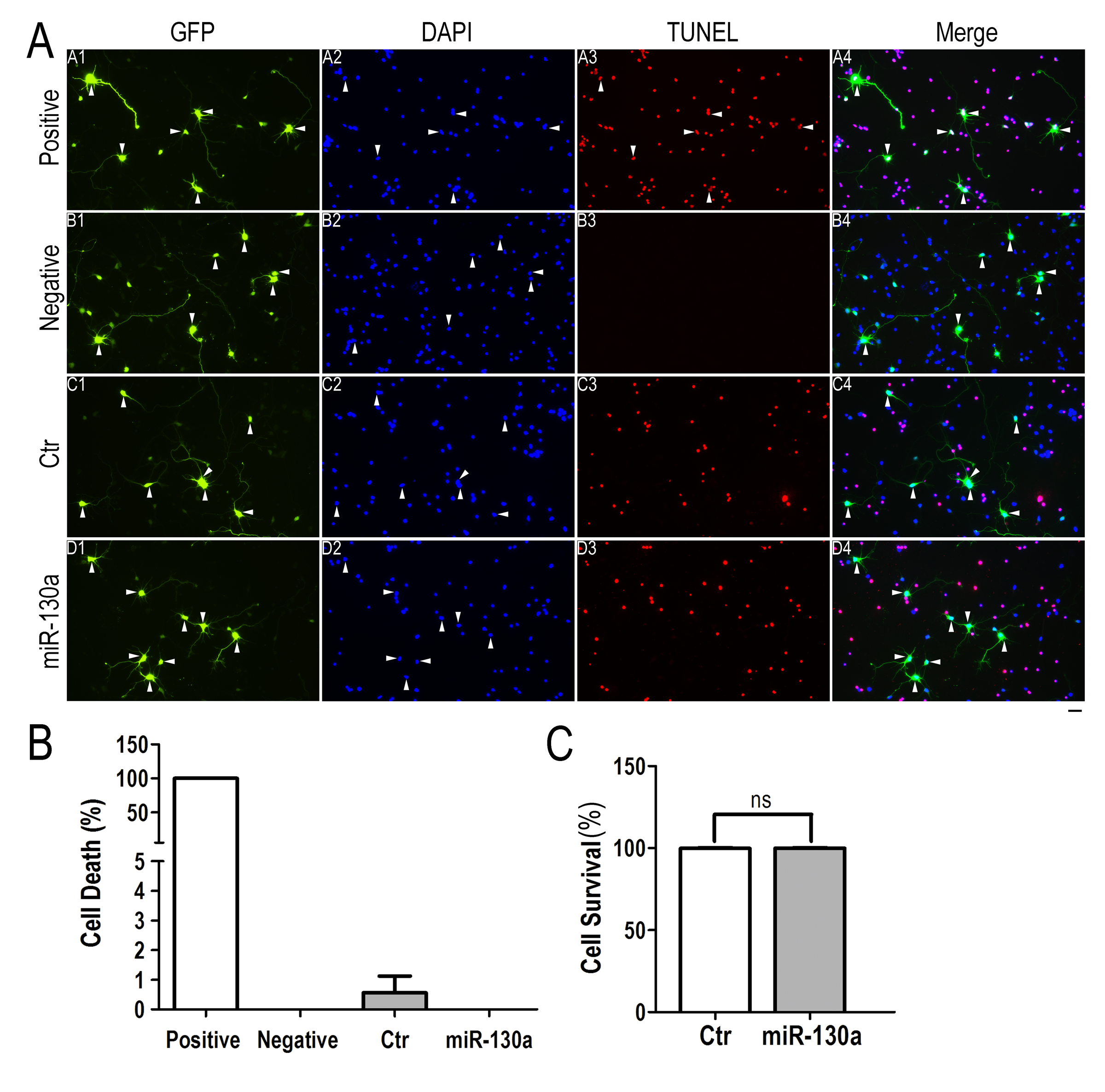

Supplement: Supplementary file 2 — Supplementary material 2 (TIFF 2321 kb) [file 13238_2016_272_MOESM2_ESM.tif]

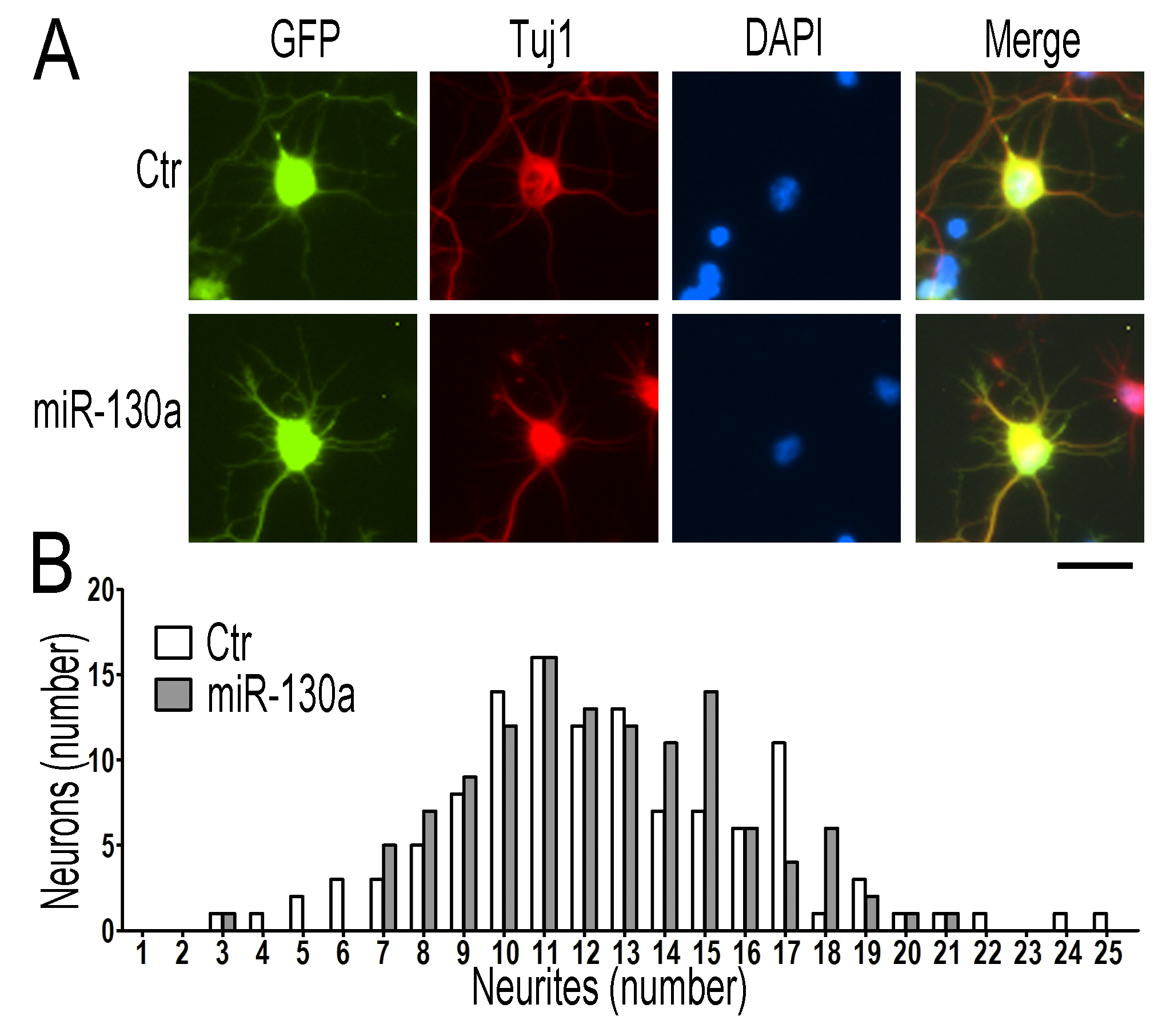

Supplement: Supplementary file 3 — Supplementary material 3 (TIFF 986 kb) [file 13238_2016_272_MOESM3_ESM.tif]
